# From “Transient Hemiopsia” to Migraine Aura

**DOI:** 10.3390/vision5040054

**Published:** 2021-11-05

**Authors:** Mark William Weatherall

**Affiliations:** Department of Neurology, Stoke Mandeville Hospital, Buckinghamshire Healthcare NHS Trust, Aylesbury HP21 8AL, UK; mark.weatherall1@nhs.net

**Keywords:** migraine, aura, hemiopsia, teichopsia, cortical spreading depression

## Abstract

This paper outlines the historical development of the concept of the visual aura of migraine, from the first comprehensive published description by the physician Hubert Airy, in 1870. Airy’s description of the phenomenon he called “transient hemiopsia” became widely copied and highly influential as a consequence of the language and images that he used in his presentation. This paper outlines the subsequent development of theories of aura from the time of Airy’s publication to the first demonstration of spreading oligaemia by Lautitzen and Olesen in the 1980s.

“Just as all operas have an overture getting the spectator ready, and a book has its foreword to inform the reader of the situation, so each migraine has its foreword, or overture… This *aura*… may show endless variations… it is mandatory… and free of charge” [1], as quoted in [2].

On 17th February 1870, the Astronomer Royal, George Biddell Airy, read a paper written by his son, Dr. Hubert Airy, to the assembled members of the Royal Society of London. It was, at that time, customary for such papers to be communicated and read by Fellows of Society. Hubert Airy’s paper, “On a distinct form of Transient Hemiopsia”, was subsequently published in the *Philosophical Transactions* of the Society [3]. Through the advocacy of contemporaries such as George Liveing, Peter Wallwork Latham, and William Gowers, Airy’s paper came to be regarded as the first definitive English language description of the visual aura of migraine. In this paper, I will discuss how Airy’s hemiopsia (a neologism meaning “half-eyesight”) became an archetype for migraine aura through the dissemination of the striking language and images that he used in his paper. I also briefly outline the development of theories of aura from the time of Airy’s publication to the first demonstration of spreading oligaemia by Lautitzen and Olesen in the 1980s.

## 1. Migraine and 19th Century British Men of Science

In his 1870 paper, Airy presented a precis of previous accounts of hemiopsia, including several published in the 19th century by eminent men of science: William Hyde Wollaston, Sir David Brewster, and Airy’s father, George. Wollaston, Brewster, and the elder Airy all seem to have been predominantly interested in their visual experiences as potentially instructive in the broader context of the increasing interest in the physical basis of light, the laws of optics, and the psychology of visual perception, all of which were current and predominant interests in 19th century British science. Hubert Airy approached the phenomena from a more purely medical point of view, commencing his account by asking, perhaps only semi-rhetorically, why members of his profession had not taken more interest in such a common and fascinating phenomenon. Wollaston believed the condition “to be far more common than is generally supposed; and I might with as much reason have expressed surprise at its having so far escaped notice, were I not aware how many facts commonly remain disregarded, merely for want of explanation”. An addendum to Wollaston’s article in the *Philosophical Magazine* noted that even in the short period between its composition and publication, he had uncovered two more cases. George Airy had compared his own experiences with those of his friends, pointing out similarities in the phenomena, but also differences, both in the triggering circumstances (one of his friends attributed them to mental anxiety or overcrowding of business), and in related symptoms (distortion of the mouth in his friend’s case, and problems with speech and memory in his own). Hubert Airy mentions at least three other people’s experiences. It is not clear whether these are patients or acquaintances, although he does present an entry from his diary in which he recorded his first ever attack, commenting that a female acquaintance was subject to these attacks of “half-blindness”. The common thread that runs through all these accounts is a degree of slight bafflement as to why such a common human experience should have essentially escaped the notice of the medical profession [4].

Rereading Airy’s account after 150 years have passed, one finds much that remains contemporary and relevant. That it does so is testament to the influence this paper has had in the subsequent understanding of migraine. It is important to remember, however, that it was by no means self-evident at the time that this would be the case. Airy was not a well-known doctor. The subject matter was not something of particular interest to the medical profession or indeed to men of science in general. It was true that eminent scientists had written about migraine aura, but they had had their own reasons for doing so, and Airy’s reasons for including those accounts have more to do with establishing the validity of the phenomenon, as with the interpretation that previous men of science had put upon it. There are perhaps three main reasons why Airy’s paper has come to be regarded as a classic: first (and perhaps foremost) the drawings of the phenomena that Airy included; second, the language that he used to describe the phenomena, and in particular his use of metaphors which subsequently became commonplace; and thirdly, the central role that subsequent neurologists, in particular the renowned William Gowers, gave his account when they came to discuss the subjective visual sensations that could be associated with migrainous headaches.

## 2. Models and Metaphor

Airy did not use the term “aura” for the phenomenon that he described. As the historian and biographer of migraine Esther Lardreau has demonstrated, “aura” was a term that had predominantly been applied to sensory symptoms that preceded an epileptic seizure; this usage continues to the present day [2]. Airy instead coined two terms for the phenomenon, the first of which was contained in the title of his paper, “On a distinct form of Transient Hemiopsia”. As befitted an alumnus of Trinity College, Cambridge, Airy did not wear his erudition lightly, and the neologism, meaning “half-eyesight”, was unusual enough for him to feel it necessary to include an explanatory footnote detailing the Greek roots of the word.

Airy’s second neologism for the phenomenon—“teichopsia”—proved more pervasive. This term became the most successful manifestation of the multiplicity and outflowing of metaphor in Airy’s paper. The term, Greek for “town-wall vision”, rested on a series of descriptions of the positive phenomena of aura being angular, like those of fortifications, dating back to a 1778 paper by the physician John Fothergill. By “fortification”, Fothergill meant the triangular pattern of the outer walls of a mediaeval town or castle seen from above, or on a plan, rather than the crenelations atop a castle wall, as some subsequently interpreted it. The eminent 19th century mathematician and astronomer Sir John Herschel was precise in comparing the phenomena to “salient and re-entering angles, bastions, and ravelins”. The 20th century neurologist Macdonald Critchley recounts how Charcot compared his own aura to “one of Marshal Vauban’s fortifications with its salients and recesses” (Vaubon was responsible for the defence of Paris in the 1870 Franco-Prussian war). The same thought appears to have suggested itself spontaneously to Hubert Airy, who in his diary in 1854 recorded that his first experience of aura was “like a fortified town with bastions all round it, these bastions being coloured most gorgeously”. Airy also compared the phenomena to a “cloud”, or a “thick liquid all alive”: “boiling”, “seething”, full of “turbulence and trembling”.

At one point, Airy calls the visual phenomenon a “strange intruder”. The “otherness” of aura—the sense that aura is something at once familiar, yet alien—is reinforced by Airy’s recurrent use of military metaphors, an increasingly common trope in medicine from the 1860s onwards, and which still pervades migraine literature (both lay and technical) to the present day. Throughout he refers to the episodes as “attacks”. He talks of “the disease” extending and spreading outwards to “invade the more distant parts of the field of vision”. Occasionally, he observes “the rudiments of a fresh attack, beginning nearly where the first began, and sometimes advancing so far as to exhibit its bastioned margin”. With this sense of being under attack comes an oppression, a horror of the experience: “I have seen a person”, writes Airy, “terribly subject to these attacks, shudder at the very name, and turn away from a drawing of the ugly sight, quite content to bear serious illness “if only the ‘half-blindness’ would keep away””. The visceral otherness of the experience proved a fertile ground for contemporary writers, who found the descriptions of fragmented vision, bodies, and consciousness a relevant symbol for the prevalent anxieties of the 19th century [5].

**Figure 1:**
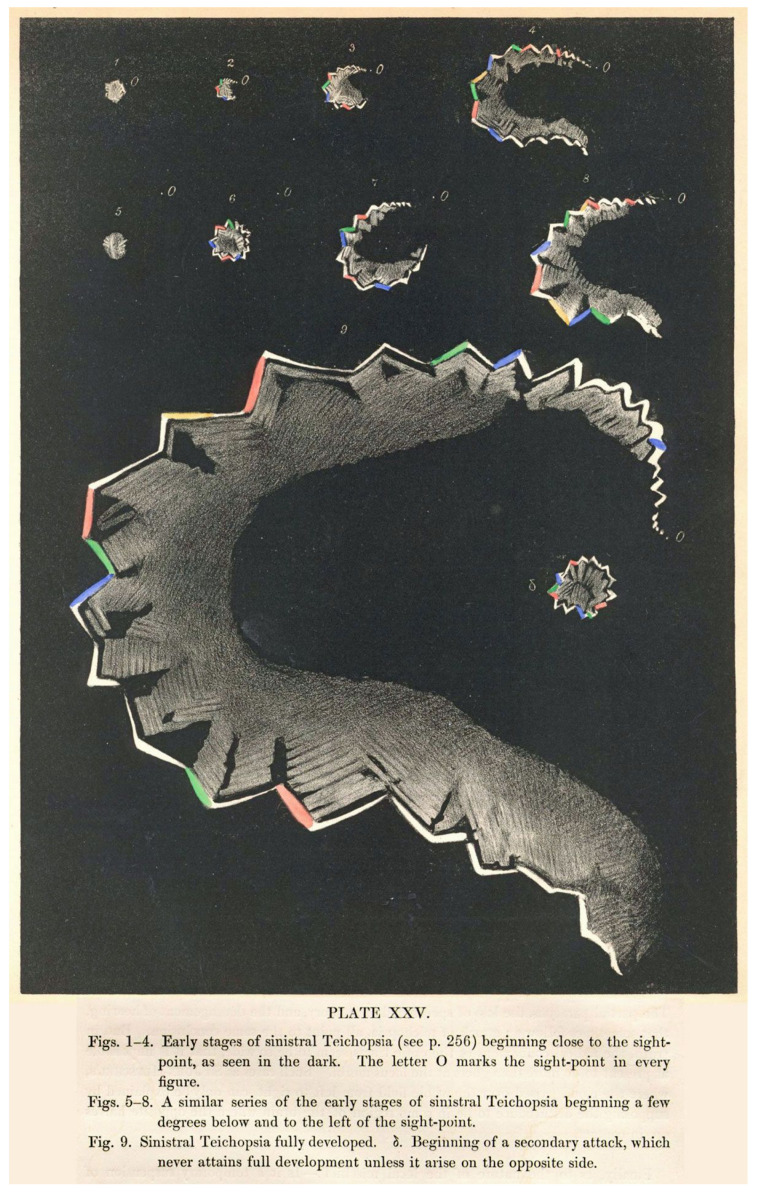
Illustrations of teichopsia from Airy’s 1870 paper for the Royal Society [3].

That Airy should show a drawing of the phenomenon to discover whether his friends and acquaintances were familiar with it is not surprising. Airy’s father had included a representation of his experiences in his short paper on the topic. Scientists were interested in constant, reproducible phenomena that could be measured, quantified, and understood. Visual aura was transient, disconcerting; it undermined the very foundations of objective record by distorting the senses. Illustrations were necessary to fix the clinical phenomena, to prevent them from decaying in the same way that formalin prevented the otherwise inevitable putrefaction of flesh. The neurologist Geoffrey Schott, writing in the journal *Brain* in 2007, pointed out the essential role of illustration in demonstrating uniformity, and providing a heuristic for the interpretation and understanding of the underlying physiological processes [6]. Schott also notes that both the Airys provided illustrations that cleverly represented the march of the phenomena; of these, it was the son’s detailed, coloured plates that were to become the archetype for migraine aura for the next 150 years (Figure 1).

When the physician Edward Liveing needed a diagram to illustrate aura in his monograph *On Megrim*, the picture he chose was Hubert Airy’s [7]. In the same year, the Cambridge professor Peter Wallwork Latham also chose Airy’s plates to illustrate the published version of his lectures on migraine [8]. Both Liveing and Latham knew the younger Airy well, and there were few other illustrations available to them; and Airy’s picture encapsulated clearly and precisely the experience that many (but not all) people had of visual aura. Increasingly, when people needed to illustrate aura, they chose the same diagrams. Steadily, inexorably, “Airy’s hemiopsia” became “teichopsia”, and then just “aura”.

**Figure 2:**
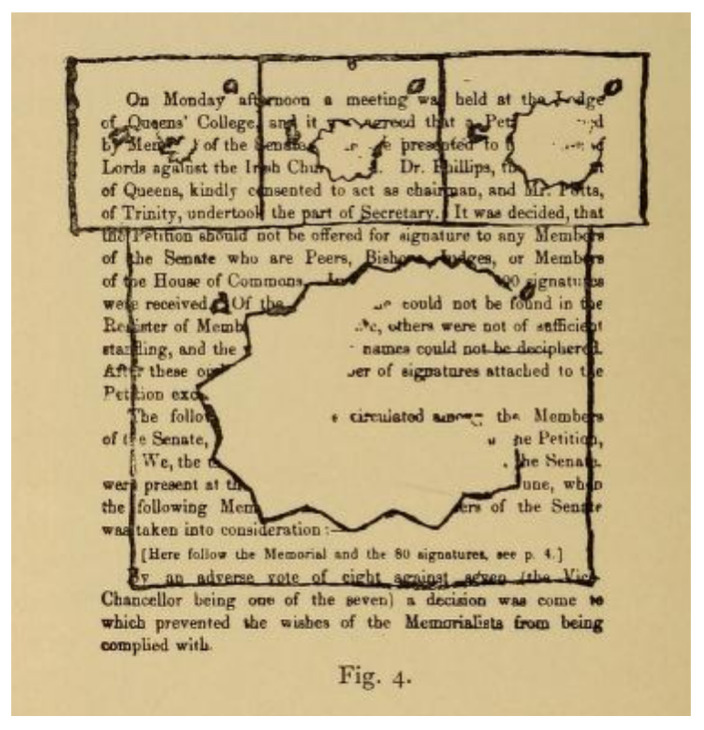
An illustration, drawn by Airy, of the effect of aura on his ability to read text. Published by Gowers in his paper on subjective visual sensations [10].

**Figure 3:**
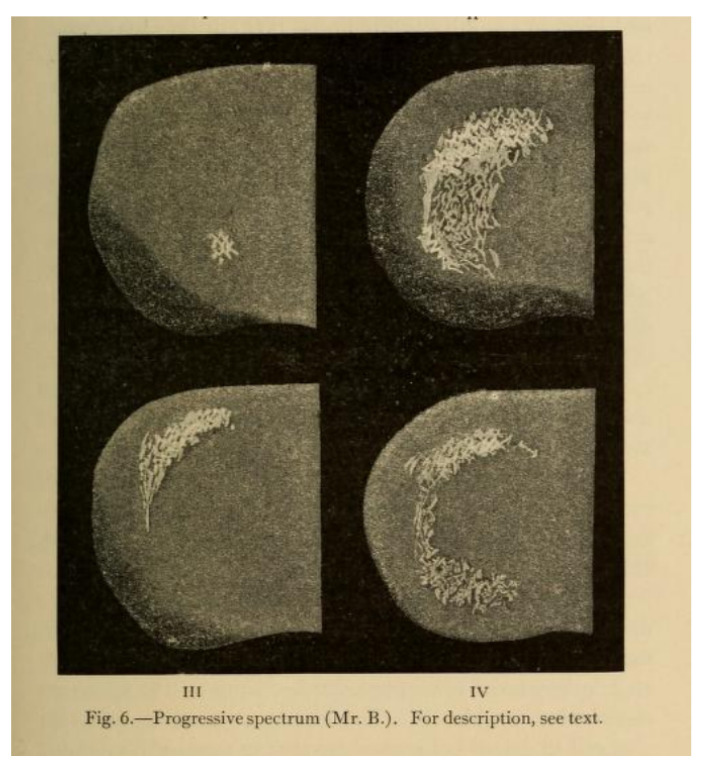
An illustration of migraine aura by Gowers’ patient, Mr. B. In the published version [10], Gowers notes his regret that “the black-and-white reproduction very imperfectly represents the original”.

The central figure in the wider dissemination of Airy’s images was the neurologist William Gowers. Gowers recognised the association of migraine with a number of typical subjective sensory symptoms (“sensory” in its wider sense included visual symptoms). Gowers cited Airy’s work in his chapters on migraine for his *Manual of Nervous Diseases*. In the *Manual*, Gowers includes a long discussion of the visual accompaniments of migraine, largely drawn from Airy’s paper, supplemented with additional cases, including that of his patient, Mr. Beck. He noted that the sensory symptoms of migraine sometimes occurred without headache [9]: “These cases are of great importance, because their nature is often misunderstood”. This section was retained and expanded in the 2nd edition, with further examples. In addition, when he chose the subject of subjective visual sensations for his Bowman Lecture to the Ophthalmological Society in 1895 [10], he asked Airy for further, previously unpublished pictures of his aura to show to the Society, along with pictures drawn for Gowers by an artist of his acquaintance who suffered from migraine (Figure 2 and Figure 3). In an address to the Westminster Medical Society in 1909 (reprinted in the *British Medical Journal*), Gowers continued to use Airy’s diagrams as the archetype of visual aura [11].

**Figure 4:**
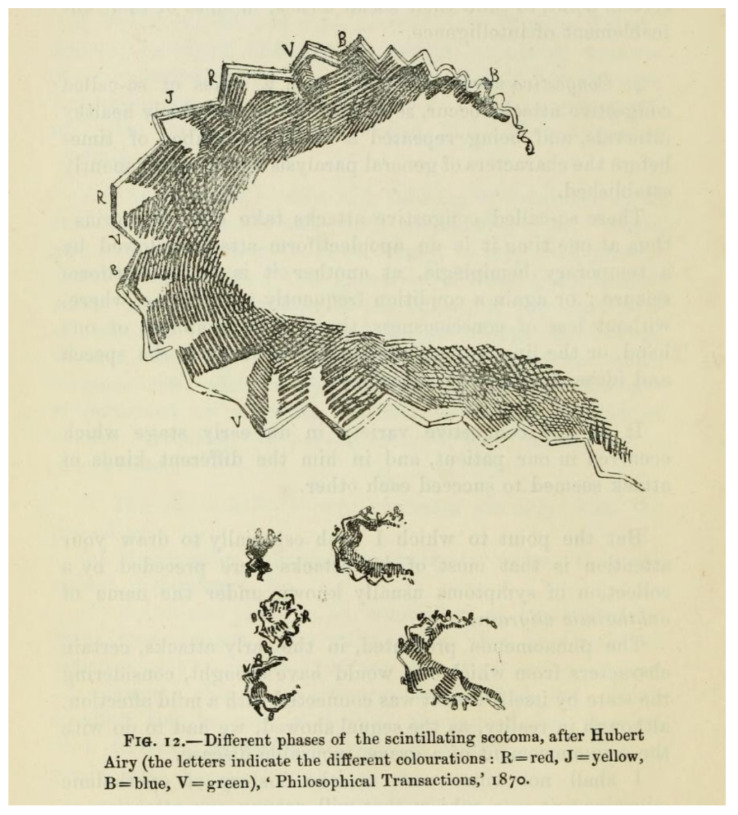
An illustration of migraine aura, “after Hubert Airy”, published in the English translation of Charcot’s lectures [12].

Across the Channel, the great French neurologist Jean-Martin Charcot, describing “migraine ophthalmique” in his *Leçons sur les maladies des système nerveux* in the 1880s [12], used language almost indistinguishable from Airy’s, and used Airy’s drawings as illustration (Figure 4).

The German ophthalmologist Julius Hirschberg cited Airy’s paper in his *Wörterbuch der Augenheilkunde* of 1887 [13]. In the following decade, his contemporary Ernst Fuchs’ account of “scintillating scotoma” in his *Text-book of Ophthalmology*, references Airy’s nomenclature, and closely follows Airy’s description of the phenomenon [14].

By the 1880s and 1890s, the term “teichopsia” started to appear in accounts of migraine in standard medical textbooks. Charles Hilton Fagge, in the first edition of his *Principles and Practice of Medicine* in 1886, reprinted portions of Airy’s paper verbatim, even whilst calling into question the usefulness of the term “teichopsia” [15]. The description of aura in the first (1890) and subsequent editions of Frederick Taylor’s *A Manual of the Practice of Medicine* is clearly based closely on Airy’s text and diagrams [16]. The most famous of all the fin de siècle textbooks, William Osler’s *Principles and Practice of Medicine*, from its first edition in 1892, stressed how, in deference to Airy’s illustrations, “the so-called fortification spectra (teichopsia)… may be illuminated with gorgeous colours” [17]. The neurologist Chris Boes has demonstrated that it is likely to be the case that Osler based the neurological portions of his textbook on Gowers’ *Manual* [18].

**Figure 5:**
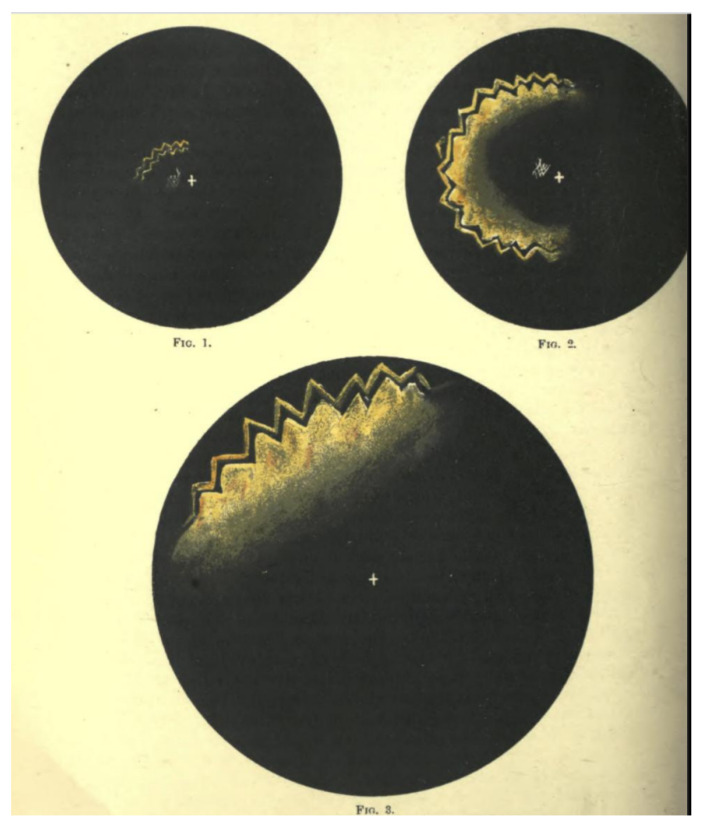
James Mackenzie’s illustrations of migraine aura, published to accompany his article on migraine for Allbutt and Rolleston’s *System of Medicine* [19].

Even where Airy’s own pictures were not used, they were quickly understood to be the standard reference for the phenomenon. In his article on migraine for the second edition of Allbutt’s *System of Medicine*, for example, the general practitioner James Mackenzie (subsequently to become renowned as a pioneer of the use of ECG technology in assessing diseases of the heart) presented an account, complete with coloured plates (Figure 5), of his own attacks of migraine, but felt it necessary to explain that, in his case, “the hues are not so strikingly iridescent as in the spectra given by Airy, the colours being mostly of a brilliant luminous yellow” [19].

Through this process of reproduction and dissemination, Airy’s images became, as the historian Katherine Foxhall has commented, an example of what Lorraine Daston and Peter Galison have termed a “working object” for migraine: a standardised exemplar that taught practitioners ‘“what is worth looking at, how it looks and perhaps most importantly how it should be looked at” [20]. Foxhall notes that, by the time of the Great War, the widespread dissemination of Airy’s images provided the background to Charles Singer’s identification of the visions of Hildegaard of Bingen as being migrainous [21].

## 3. Neural and/or Vascular? Theories of Aura 1870–1985

Hippocrates wrote of a man who saw “something shining before him like a light, usually in part of the right eye”, who then developed “a violent pain that supervened in the right temple, then in all the head and neck”, which settled down after he vomited. Exactly how these phenomena were related was not understood. Advances in the understanding of cerebral localisation in the 19th century did not make things any clearer. It was well known that the visual centres of the brain were in the occipital lobes, but it was not at all clear where pain was located. By the time Airy published his description of aura, the likeliest candidate was an area at the base of the brain called the optic thalamus. Liveing considered the phenomena of migraine to be due to a “nerve-storm” passing through this part of the brain. Latham, in common with leading continental scientists, contended that aura was due to constriction of the blood supply to the visual area of the brain, and the succeeding headache due to expansion of the same vessels, possibly mediated through the sympathetic nervous system. This vascular theory was simple to understand and popular; it was the most widely held explanation for aura for over 100 years; but had its obvious problems, as William Gowers pointed out from the beginning. Constriction of the blood vessels should restrict blood supply to a particular area of the brain and stop that area of the brain working. If this affected the visual area of the brain, it should cause a loss of vision. The sparkling scintillations characteristic of aura were difficult to explain, as was the progression of symptoms across the visual fields. It was also clear that, in many cases, headache started before aura had completely resolved, a fact which could not be explained by the vascular theory. Gowers favoured a neural conception of the aura process, though he did not think that Liveing’s conception of a nerve-storm was accurate or useful [22]. Gowers (and other British neurologists, such as John Hughlings Jackson) recognised the analogies between migraine and epilepsy, though Gowers was at pains to distinguish the visual sensations seen in epilepsy (“extremely brief… followed by loss of consciousness or convulsion”) from those of migraine (“deliberate, slow in evolution, occupying more minutes than… seconds… followed… by a headache lasting hours”) [10,23]. Late in life, in his 1909 lecture to the Westminster Medical Society, Gowers seemed to be reassessing the possibility of a vascular process at work, though the published account is unclear [11]: “Most of the unilateral premonitions may be explained by the limitation of the disturbance to one hemisphere, by whatever it is excited, arterial spasm or whatever may be productive of the activity of function”.

The difficulty of explaining aura in straightforward vascular terms may explain why the neurologist Harold Wolff, who was the first to demonstrate how blood flow in extracranial blood vessels changed during migraine headache (and how ergotamine impacted upon this process), struggled to explain the phenomena, only devoting eight out of the 600 pages that made up the first edition of his monograph *Headache and Other Head Pain* in 1948 [24]. Wolff hypothesised that the aura was due to vasoconstriction, citing cases where aura had been abolished by the inhalation of the vasodilator amyl nitrate. This substance had been used to treat migraine since the beginning of the century: in his article for Allbutt’s *System of Medicine*, James Mackenzie reported having tried, unsuccessfully, to abort his own auras with it [19].

In 1940, the possibility that aura was a primarily neural process, consisting of a wave of excitation and then inhibition, was raised again by the visual physiologist Karl Lashley, who published meticulous descriptions of his own visual aura [25]. In 1958, the Canadian neurologist PM Milner proposed Leão’s spreading depression as a candidate process by which such a wave could propagate across the surface of the brain [26], a possibility that Leão himself had mentioned briefly in a paper in 1945 [27]. Cortical spreading depression (CSD) was a neurophysiological phenomenon first described by the Argentinian neurophysiologist in the 1940s, in which a wave of excitation spread slowly across the surface of the brain, followed by a longer period of inhibition, before normal function was restored [28].

It was not until the early 1980s, however, that a physiological process was firmly linked to the experience of migraine aura in humans. It was known that ~50% of patients who were prone to visual aura might have an attack after carotid angiography. The Danish neurologists Martin Lauritzen and Jes Olesen used this as an opportunity to study cerebral circulation in the initial phases of a migraine attack by injecting radioactive xenon-133 into the carotid arteries. They found that, in their migraine patients, this caused a decrease in regional cerebral flow that began in the posterior part of the brain and progressed anteriorly in a slow wave, independent of the territories that were supplied by the large cerebral arteries. They theorised, therefore, that aura was the experience of CSD: a wave of neural excitation initiated in the posterior part of the brain, progressing anteriorly with a constant speed of approximately 2–3 mm/min, and that it was the transient absence of nervous activity that *caused* reduced blood flow to the affected areas; that is, spreading oligaemia, and by extension aura, was mediated by a primary disturbance of nerve cell function, rather than being primarily vascular [29].

Subsequent research studies confirmed Lauritzen and Olesen’s original findings, and replicated them by other means, such as functional MRI scanning [30]. In addition, CSD provides an explanation for the other manifestations of aura: if it starts elsewhere in the brain, or spreads beyond the occipital lobes, it can affect regions that deal with sensation, language, motor function, emotion, and so on. Why migraine aura has a particular predilection for the occipital lobes, and why it in most people it fizzles out before it reaches other parts of the brain, are questions that remain largely unanswered.

## 4. Conclusions

Writing in his 2012 book *Hallucinations*, the neurologist Oliver Sacks recalled the intellectual excitement and emotional engagement of seeing migraine patients in a migraine clinic in New York in the mid-1960s [31].

“I was fascinated by the range of symptoms and phenomena that could occur in migraine attacks. These attacks often included an aura, a prodrome in which aberrations of perception and even hallucinations occurred. They were entirely benign and would only last a few minutes, but those few minutes provided a window onto the functioning of the brain and how it could break down and then reintegrate. In this way, I felt, every attack of migraine opened out into an encyclopedia of neurology”.

Sacks, himself a lifelong migraine sufferer, went on to write a book on migraine which, whilst it has its detractors, remains the richest modern monograph on the subject. Sacks places aura front and centre in his book, for the reasons, both intellectual and emotional, outlined in the quote above. Sacks also praises the Victorian pioneers of the English language migraine literature, especially Liveing, who he sees as his direct intellectual ancestors.

As I hope I have shown in this paper, those of us who see patients with migraine remain indebted to the doctors who carefully and meticulously detailed the clinical phenomena of the condition. Even now, an internet search for pictures of migraine aura will bring up, nestled amongst photoshopped representations of scintillating scotomata, Airy’s original plates from his 1870 paper, testament to the enduring power of his images. “Teichopsia” nestles securely in the medical dictionaries and textbooks, demonstrating the ability of metaphor to pervade and persist in the medical literature. Airy’s aura continues to be, in many ways, the archetype of migraine aura.

## References

[1] Soula E. (1884). Contribution à L’Etude de la Migraine, Migraine et Arthritisme. Thèse de Médecine.

[2] Lardreau E. (2007). The difference between epileptic auras and migrainous auras in the 19th century. Cephalalgia.

[3] Airy H. (1870). On a Distinct Form of Transient Hemiopsia. Phil. Trans. R. Soc. Lond..

[4] Eadie M.B. (2009). Hubert Airy, contemporary men of science and the migraine aura. J. R. Coll. Physicians Edinb..

[5] Zehentbauer J.Y. (2015). Scintillating Scotoma. Migraine, Aura, and Perception in European Literature, 1860–1900. Ph.D. Thesis.

[6] Schott G.D. (2007). Exploring the visual hallucinations of migraine aura: The tacit contribution of illustration. Brain.

[7] Living E. (1873). On Megrim, Sick-headache, and Some Allied Disorders. A Contribution to the Pathology of Nerve-Storms.

[8] Latham P.W. (1873). On Nervous or Sick Headache, Its Varieties and Treatment.

[9] Gowers W.R. (1886). A Manual of Diseases of the Nervous System.

[10] Gowers W.R. (1904). Subjective Sensations of Sight and Sound, Abiotrophy and Other Lectures.

[11] Gowers W.R. (1909). An Address ON THE PRODROMAS OF MIGRAINE: Delivered before the Westminster Division of the British Medical Association. Br. Med. J..

[12] Charcot J.-M. (1889). Clinical Lectures on Diseases of the Nervous System Delivered at the Infirmary of the Salpètrière.

[13] Hirschberg J. (1887). Wörterbuch der Augenheilkunde.

[14] Fuchs E. (1892). Text-Book of Ophthalmology.

[15] Fagge C.H. (1886). Principles and Practice of Medicine.

[16] Taylor F. (1890). A Manual of the Practice of Medicine.

[17] Osler W. (1892). The Principles and Practice of Medicine, Designed for the Use of Practitioners and Students of Medicine.

[18] Boes C.J. (2016). Gowers and Osler: Good friends ‘all through’. J. R. Coll. Physicians Edinb..

[19] Mackenzie J., Allbutt T.C., Rolleston H.R. (1908–1911). Migraine. A System of Medicine by Many Writers; Diseases of the Brain and Mental Diseases.

[20] Daston L., Galison P. (2010). Objectivity.

[21] Foxhall K. (2014). Making Modern Migraine Medieval: Men of Science, Hildegard of Bingen and the Life of a Retrospective Diagnosis. Med. Hist..

[22] Weatherall M.W. (2012). The migraine theories of Liveing and Latham: A reappraisal. Brain.

[23] Gowers W.R. (1906). Clinical lectures on the borderland of epilepsy. III.-MIGRAINE. Br. Med. J..

[24] Wolff H.G. (1948). Headache and Other Head Pain.

[25] Lashley K.S. (1941). Patterns of cerebral integration indicated by the scotoma of migraine. Arch. Neurol. Psychiatry.

[26] Milner P.M. (1959). Note on a possible correspondence between the scotomas of migraine and spreading depression of Leão. Electroencephalogr. Clin. Neurophysiol.

[27] Leão A.A.P., Morrison R.S. (1945). Propagation of spreading cortical depression. J. Neurophysiol..

[28] Leão A.A.P. (1944). Spreading depression of activity in cerebral cortex. J. Neurophysiol..

[29] Olesen J., Larsen B., Lauritzen M. (1981). Focal hyperemia followed by spreading oligemia and impaired activation of rCBF in classic migraine. Ann. Neurol..

[30] Tfelt-Hansen P.C. (2010). History of migraine with aura and cortical spreading depression from 1941 and onwards. Cephalalgia.

[31] Sacks O. (2012). Hallucinations.

